# Supplementary figures and images for: A mitochondrial carrier transports glycolytic intermediates to link cytosolic and mitochondrial glycolysis in the human gut parasite Blastocystis
Source: eLife. 2024 May 23;13:RP94187. doi: 10.7554/eLife.94187 (PMC11115451; doi:10.7554/eLife.94187)

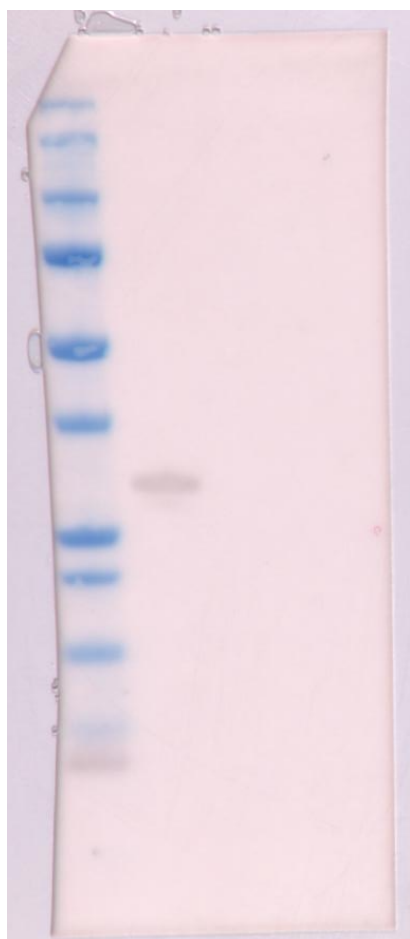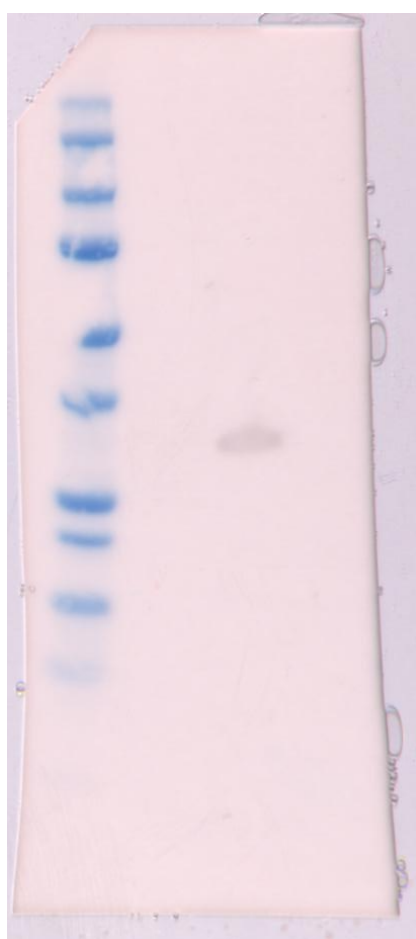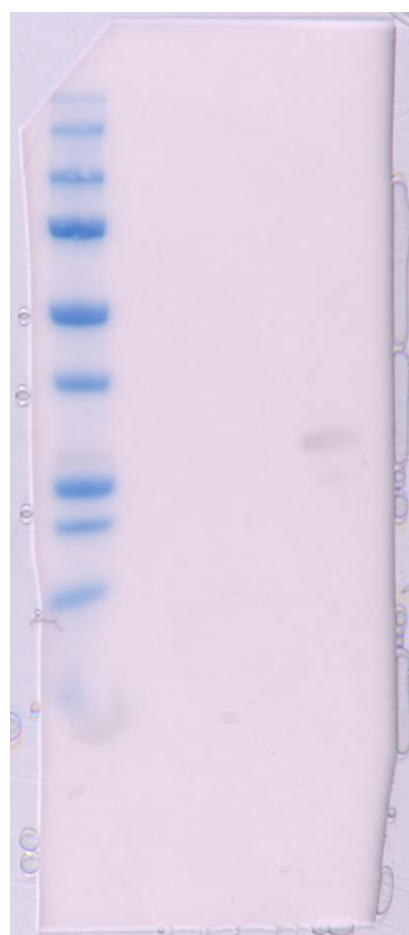

Supplement: Figure 2—source data 1. [file elife-94187-fig2-data1.pdf]

**B**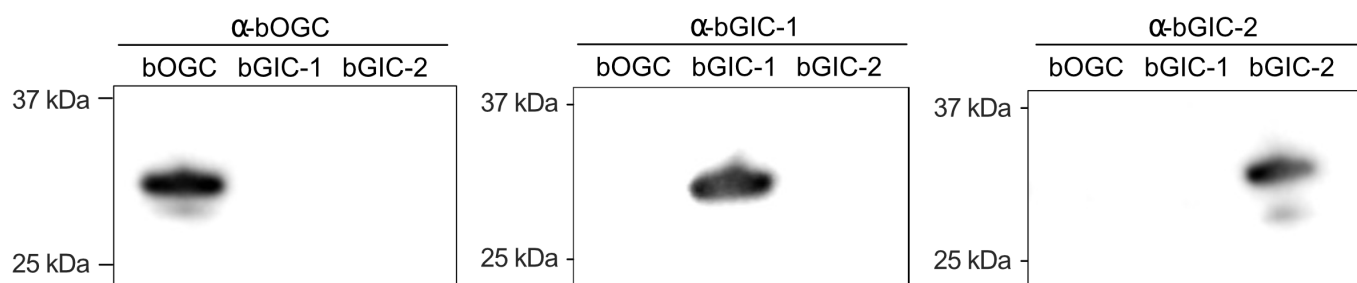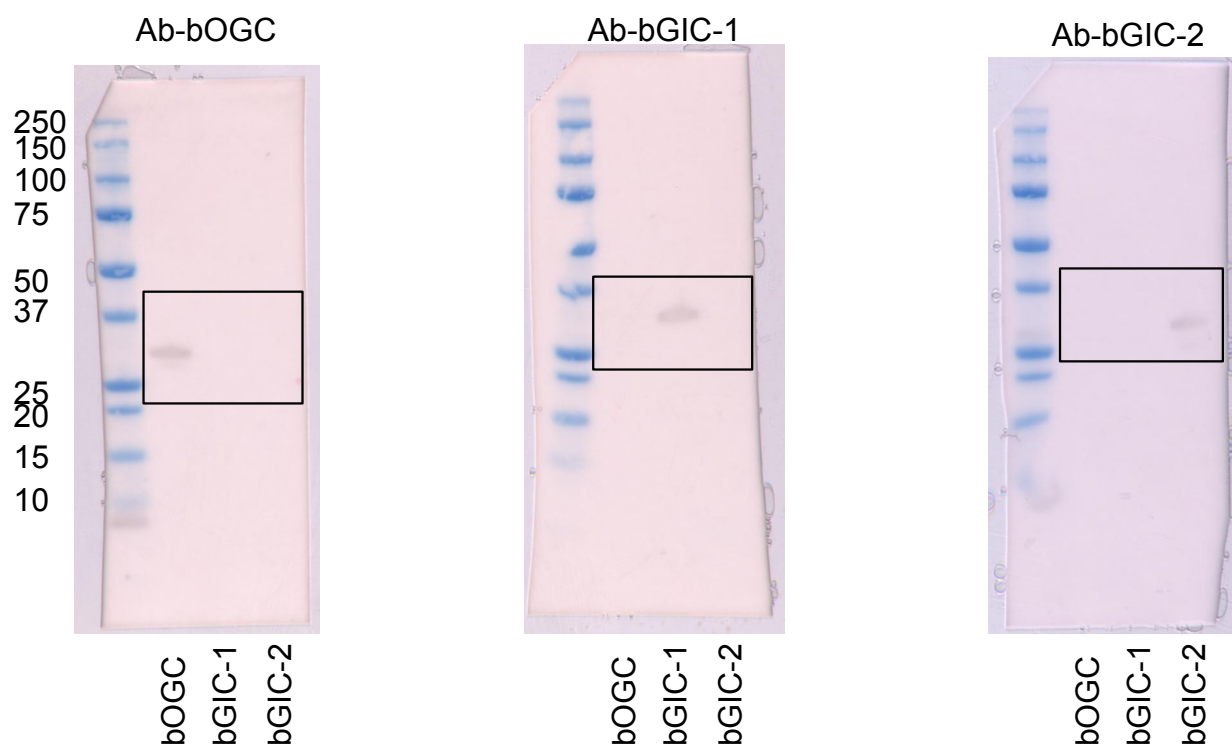

Supplement: Figure 2—source data 2. [file elife-94187-fig2-data2.pdf]

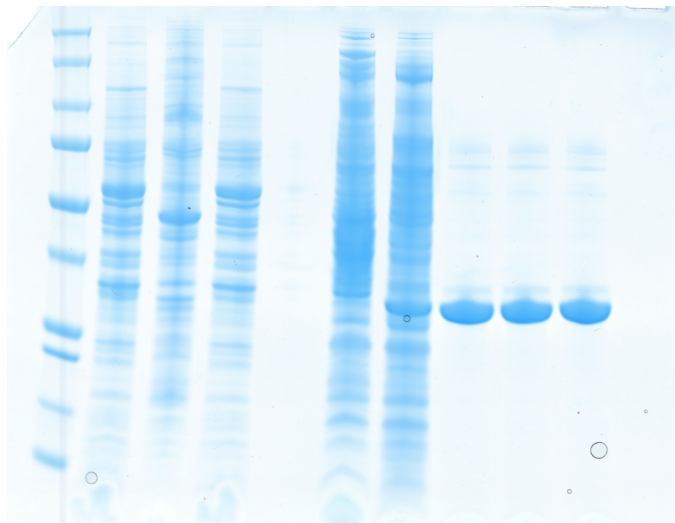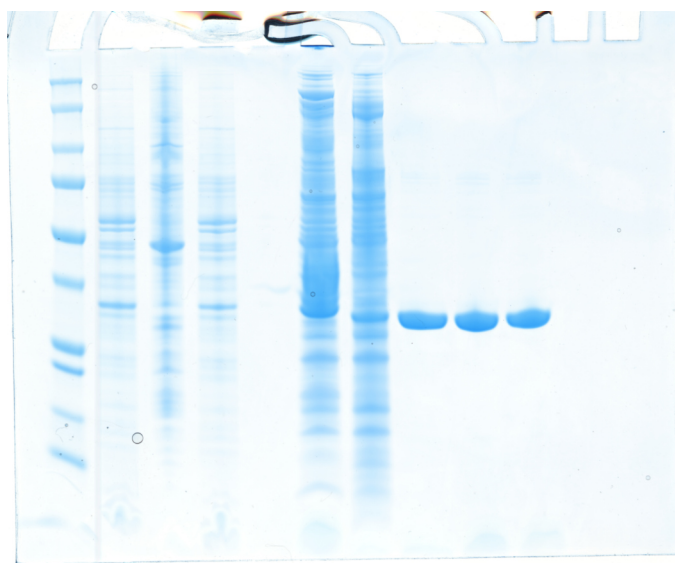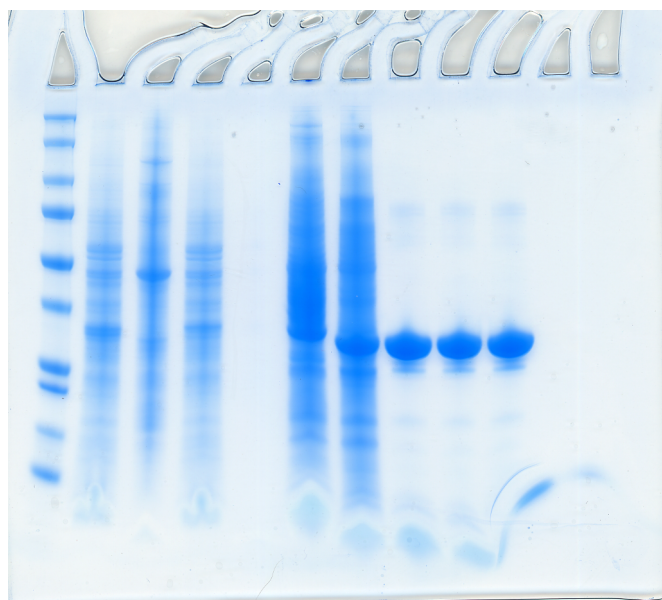

Supplement: Figure 3—source data 1. [file elife-94187-fig3-data1.pdf]

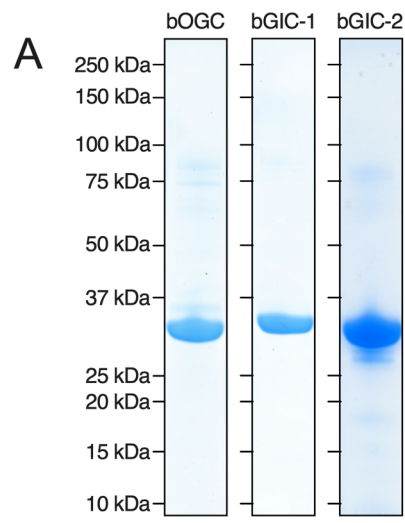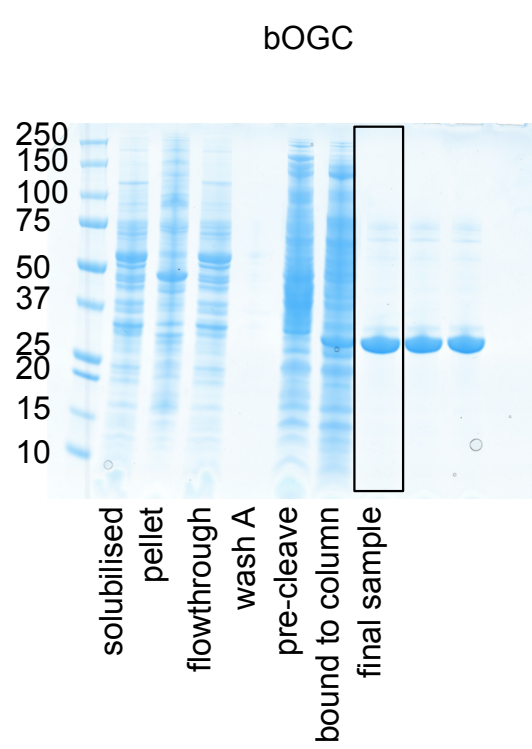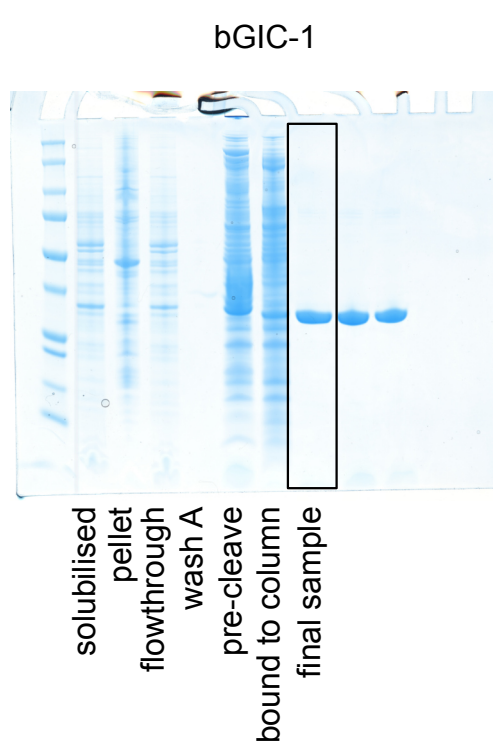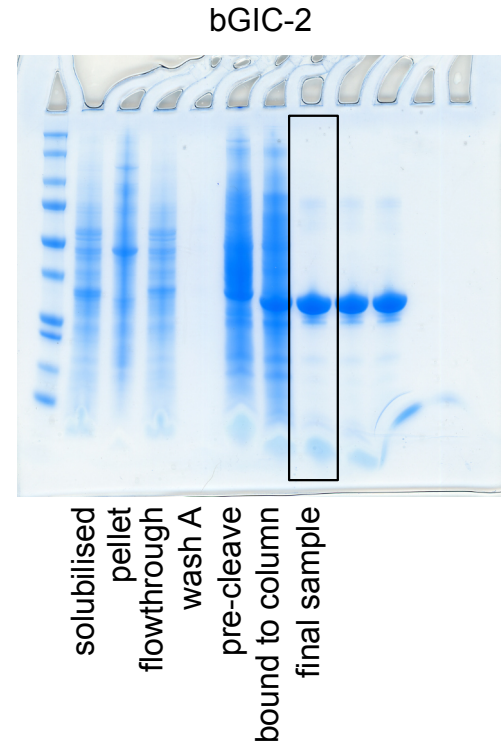

Supplement: Figure 3—source data 2. [file elife-94187-fig3-data2.pdf]
